# Metabolic responses in blood-stage malaria parasites associated with increased and decreased sensitivity to PfATP4 inhibitors

**DOI:** 10.1186/s12936-023-04481-x

**Published:** 2023-02-14

**Authors:** Shivendra G. Tewari, Rubayet Elahi, Bobby Kwan, Krithika Rajaram, Suyash Bhatnagar, Jaques Reifman, Sean T. Prigge, Akhil B. Vaidya, Anders Wallqvist

**Affiliations:** 1 Department of Defense Biotechnology High Performance Computing Software Applications Institute, Telemedicine and Advanced Technology Research Center, U.S. Army Medical Research and Development Command, Fort Detrick, MD USA; 2 The Henry M. Jackson Foundation for the Advancement of Military Medicine, Inc., Bethesda, MD USA; 3 Department of Molecular Microbiology and Immunology, Johns Hopkins University, Baltimore, MD USA; 4 Center for Molecular Parasitology, Department of Microbiology and Immunology, Drexel University College of Medicine, Philadelphia, PA USA; 5 Present Address: Office of Vaccines Research and Review, Center for Biologics Evaluation and Research, U.S. Food and Drug Administration, Silver Spring, MD USA

**Keywords:** Drug resistance, Genome-scale metabolic network model, Metabolomics, Na^+^ homeostasis, *Plasmodium falciparum*, Transcriptomics

## Abstract

**Background:**

Spiroindolone and pyrazoleamide antimalarial compounds target *Plasmodium falciparum* P-type ATPase (PfATP4) and induce disruption of intracellular Na^+^ homeostasis. Recently, a PfATP4 mutation was discovered that confers resistance to a pyrazoleamide while increasing sensitivity to a spiroindolone. Transcriptomic and metabolic adaptations that underlie this seemingly contradictory response of *P. falciparum* to sublethal concentrations of each compound were examined to understand the different cellular accommodation to PfATP4 disruptions.

**Methods:**

A genetically engineered *P. falciparum* Dd2 strain (Dd2^A211V^) carrying an Ala211Val (A211V) mutation in PfATP4 was used to identify metabolic adaptations associated with the mutation that results in decreased sensitivity to PA21A092 (a pyrazoleamide) and increased sensitivity to KAE609 (a spiroindolone). First, sublethal doses of PA21A092 and KAE609 causing substantial reduction (30–70%) in Dd2^A211V^ parasite replication were identified. Then, at this sublethal dose of PA21A092 (or KAE609), metabolomic and transcriptomic data were collected during the first intraerythrocytic developmental cycle. Finally, the time-resolved data were integrated with a whole-genome metabolic network model of *P. falciparum* to characterize antimalarial-induced physiological adaptations.

**Results:**

Sublethal treatment with PA21A092 caused significant (*p* < 0.001) alterations in the abundances of 91 *Plasmodium* gene transcripts, whereas only 21 transcripts were significantly altered due to sublethal treatment with KAE609. In the metabolomic data, a substantial alteration (≥ fourfold) in the abundances of carbohydrate metabolites in the presence of either compound was found. The estimated rates of macromolecule syntheses between the two antimalarial-treated conditions were also comparable, except for the rate of lipid synthesis. A closer examination of parasite metabolism in the presence of either compound indicated statistically significant differences in enzymatic activities associated with synthesis of phosphatidylcholine, phosphatidylserine, and phosphatidylinositol.

**Conclusion:**

The results of this study suggest that malaria parasites activate protein kinases via phospholipid-dependent signalling in response to the ionic perturbation induced by the Na^+^ homeostasis disruptor PA21A092. Therefore, targeted disruption of phospholipid signalling in PA21A092-resistant parasites could be a means to block the emergence of resistance to PA21A092.

**Supplementary Information:**

The online version contains supplementary material available at 10.1186/s12936-023-04481-x.

## Background

Malaria caused by *Plasmodium* spp. is a disease with ancient roots [1]. Of the five *Plasmodium* species that infect humans, *Plasmodium falciparum* causes the most severe form of malaria. According to the World Health Organization in 2020 [2], malaria caused 241 million infections and 627,000 deaths worldwide. Of these, *P. falciparum* was responsible for 98% of the deaths [2].

Artemisinin is the current frontline drug for treating *P. falciparum* malaria. However, the emergence of drug resistance is causing artemisinin-based therapies to fail in Southeast Asia [3] and Africa [4]. To enhance treatment efficacy and, potentially, suppress drug resistance development, the current focus is on developing artemisinin-based therapies combining artemisinin with two or more drugs [5]. However, due to the recurring loss of anti-malarial drugs to resistance, there will always be a continuous need to identify novel drugs and targets.

The search for anti-malarial drugs with novel modes of action led to the identification of PfATP4-targeting compounds, such as spiroindolones [6], pyrazoleamides [7], and SJ733 [8, 9], that disrupt Na^+^ homeostasis in *P. falciparum*. Prolonged exposures to Na^+^ homeostasis disruptors cause mutations in the PfATP4 protein conferring drug-resistance in subsequent progeny [6, 7, 9–11]. Interestingly, Flannery et al. [11] found that exposing the chloroquine-resistant Dd2 strain to sublethal concentrations of pyrazoleamide compound GNF-Pf4492 for 70 days results in a Ala211Thr (A211T) mutation in PfATP4 that is responsible for resistance to GNF-Pf4492, but increases susceptibility to KAE609 (cipargamin), a spiroindolone compound.

In another study [12], continuous pressure of Dd2 strain with PA21A092 resulted in a resistant parasite line with Ala211Val (A211V) mutation in the PfATP4. These parasites, while resistant to PA21A092, are also hypersensitive to KAE609. Using a CRISPR-Cas9 approach, Bhatnagar [12] confirmed that a A211V mutation in PfATP4 of Dd2 strain is sufficient to recapitulate the increased sensitivity to KAE609 compound and decreased sensitivity to PA21A092 compound.

In this work, the goal was to characterize metabolic responses in *P. falciparum* parasites exhibiting increased resistance and sensitivity against Na^+^ homeostasis disruptor compounds. Dd2 parasites carrying the A211V mutation in the PfATP4 protein (Dd2^A211V^) were cultured in the presence of sublethal doses of PA21A092 or KAE609. At the identified doses of PA21A092 (or KAE609), a 30–70% inhibition of Dd2^A211V^ replication was observed during the second intraerythrocytic development cycle (IDC). Over the course of the first IDC, transcriptomic and metabolomic data were collected from Dd2^A211V^-infected erythrocyte cultures in the presence of sublethal doses of PA21A092 or KAE609. Quantitative analyses were performed to identify significant differences in gene transcription and metabolite abundances in treated parasite cultures. The time-resolved data were then integrated with a genome-scale metabolic network model of *P. falciparum* [13–16] to predict PA21A092-induced and KAE609-induced metabolic adaptations during the first IDC.

## Methods

### Determination of EC_50_ values for PA21A092 and KAE609

The phenotype of Dd2^A211V^ parasites was determined by performing growth inhibition assays using ^3^H-hypoxanthine (Perkin Elmer Inc., Waltham, MA) incorporation. Alanine synchronization was used to produce ring-stage parasites, as previously described [7]. The parental (Dd2) and mutant (Dd2^A211V^) parasite cultures were seeded in triplicate in 96-well plates at 1% parasitaemia and 3% haematocrit (HCT) containing 100 μL of low hypoxanthine Roswell Park Memorial Institute (RPMI) 1640 medium (Gibco, Gaithersburg, MD) and exposed to various doses of PA21A092 (or KAE609) for 48 h. At the 24 h time point, 3 μL of ^3^H-hypoxanthine were added to each well, and the growth of the parasites was assessed using incorporation of ^3^H-hypoxanthine over the remaining 24 h. The plates were frozen at − 80 °C to stop growth and lyse the cells. The lysates were transferred to EasyTabC glass fibre filters (Perkin Elmer Inc., Waltham, MA) using a Filtermate cell harvester, and scintillation counts were measured using Microscint-O in a Topcount NXT Beta Counter (Packard/ Perkin Elmer Inc., Waltham, MA). PA21A092 was synthesized in the Vaidya lab [7], and KAE609 was acquired from the Medicines for Malaria Venture. The 50% inhibitory concentration (EC_50_) of each compound was estimated by performing least-square fits to the experimental data.

### Determination of sub-lethal PA21A092 and KAE609 concentrations for sample collection

Next, drug concentrations were determined that would result in 30–70% growth inhibition in the high-parasitaemia conditions required for sample collection. To this end, a serial magnetic purification method was used to derive highly synchronized ring stage (2–6 h post-invasion) parasites at 5% parasitaemia and 2% HCT. These parasites were maintained for 40 h in a 96-well plate in the presence of different concentrations of PA21A092 (230–690 nM) or KAE609 (4.1–111 pM). For both compounds, 0.1% DMSO was used as the vehicle control and 1 µM chloroquine served as a positive control. Following 40 h of drug treatment, the parasite cultures were diluted 1:10 in fresh compound-free medium and cultured for ~ 30 h at 37 °C to give viable parasites ample opportunity to replicate. Parasitaemia was quantified by flow cytometry using SYBR-Green I (Invitrogen, Waltham, MA) and an Attune NxT flow cytometer (ThermoFisher Scientific Inc., Waltham, MA), as described previously [16, 17].

### Parasite culture and sample collection

Previously described methods and conditions [14–18] were used to generate 300 mL cultures of Dd2^A211V^ parasites maintained at a high level of synchrony (within a 4 h window of the developmental cycle) using successive rounds of magnetic purification. Parasite cultures were maintained in RPMI, including 20 mM HEPES, 12.5 µg/mL hypoxanthine, 25 µg/mL gentamicin, 0.5 µM R-lipoic acid, 0.3% sodium bicarbonate, and 0.5% AlbuMAX II (Life Technologies, Inc., Carlsbad, CA) with either 30 pM KAE609 or 400 nM PA21A092. Quadruplicate cultures (75 mL each) were created for each drug and similar uninfected control cultures with the same red blood cells, medium and drug concentrations were maintained in parallel.

Quadruplicate samples were collected from the infected and uninfected control cultures every 8 h until the 48 h time point. Briefly, 10.5 mL of parasite culture were collected from each quadruplicate culture flask every 8 h and immediately pelleted by centrifugation at 400Xg for 5 min. After the media were aspirated, 100 µL of the cell pellets were moved to 1.5 mL tubes for metabolomic profiling. Samples collected at 0, 8, 16, 24, 32, and 40 h from both the infected and uninfected control cultures were sent to Metabolon, Inc. (Durham, NC). Samples of 50 µL were also collected from the infected culture at the 0-, 8-, 16-, 24-, 32-, 40-, and 48-h time points and were sent to the Johns Hopkins Microarray core for transcriptomic profiling using Agilent microarray chip AMADID 037237 (Agilent Technologies, Inc., Santa Clara, CA). The reader is referred to [14–18] for more details of the sample-collection methods. The transcriptomic data were deposited in the National Center for Biotechnology Information Gene Expression Omnibus repository (GEO accession number: GSE218998), and the metabolomic data, as provided by Metabolon, Inc., are presented in Additional file 6: Table S1.

### Global analyses of transcriptomic and metabolomic data

For dimension reduction of transcriptomic data, MATLAB’s built-in function *tsne* implementing the t-Distributed Stochastic Neighborhood Embedding (t-SNE) algorithm was used [19]. Prior to t-SNE, a log_2_ transformation of the absolute raw values of the transcriptomic data was performed. The Mersenne Twister algorithm was used for generating random numbers for the stochastic algorithm. For comparison of the effects of PA21A092 and KAE609 on IDC progression of *P. falciparum*, Pearson correlation coefficients were computed between time-resolved transcriptomic data from KAE609-treated and PA21A092-treated Dd2^A211V^ with previously reported hourly sampled transcriptomic data from Dd2 [20] to identify the point of maximum correlation between the datasets. As a positive control of the pyrazoleamide treatment, transcriptomic data from untreated and PA21A092-treated Dd2 parasites were also included [16].

A previously published method [16] was used to compute average fold changes (FC_gene_) in *Plasmodium* gene expression in the presence of sublethal doses of KAE609 or PA21A092. For identification of statistically significant changes in gene abundances due to a compound, a two-way analysis of variance (ANOVA) comparing mean steady-state expression abundance of a gene in untreated and treated conditions was employed. The Benjamini method [21] was used to adjust *p*-values that may lead to erroneous rejection of the null hypothesis.

Because ring-stage parasites are metabolically less active than the late-stage parasites [18], average fold change in abundance of each metabolite (FC_40/8_) at 40 h (late-stage parasite) relative to 8 h (ring-stage parasite) was computed. By comparing the FC_40/8_ values of each metabolite from PA21A092-treated parasite cultures or (KAE609-treated cultures) with untreated parasite cultures, the effect of PA21A092 (or KAE609) on net metabolite accumulation or consumption during the IDC was quantified. For computing the FC_40/8_ values, the raw count of each robustly detected metabolite (> 1000 raw counts) in each sample was first normalized using its protein concentration determined by the Bradford method (Additional file 6: Table S1). For incorporation of the effect of inter-sample variability on the FC_40/8_ value, 10,000 random samples were generated, using the MATLAB built-in function *bootstrp*, from the abundances detected in the quadruplicate samples at the 8 h and 40 h time points of each metabolite. A fold change in average abundance of each metabolite was computed for all the bootstrap samples, i.e., ${\text{F}}{\text{C}}_{\text{i}}^{40/8}=\left\{{\text{m}}_{\text{i}}^{40}/{\text{m}}_{\text{i}}^{8}: \, \forall i \in {\text{N}}\right\}$. Here, N denotes the total number of bootstrap samples, ${\text{m}}_{\text{i}}^{8}$ and ${\text{m}}_{\text{i}}^{40}$, respectively, represent the average abundance of a metabolite in the ith bootstrap sample at the 8-h and 40-h time points of the experiment. Finally, FC_40/8_ of a metabolite was computed as:

1 $$ {{\text{FC}}_{{40/8}} {\mkern 1mu}  = {\mkern 1mu} \frac{1}{{\text{N}}}\sum\limits_{{{\text{j}} = 1}}^{{\text{N}}} {{\text{FC}}_{{\text{j}}}^{{{{40} \mathord{\left/ {\vphantom {{40} 8}} \right. \kern-\nulldelimiterspace} 8}}} } } $$

To quantify net accumulation or depletion in abundance of a metabolite over time, the method described by Tewari et al. [15, 16] was used to compute average fold change in abundance of each metabolite relative to its abundance at 0 h (FC_0h_).

### Predicting effects of PA21A092 and KAE609 treatment on *P. falciparum* metabolism

With the methods described thus far, the effects of PA21A092 and KAE609 on abundances of specific gene transcripts and metabolites can be characterized. However, an increase in expression of a gene need not lead to an increase in flux of the encoded enzyme [22] because an organism’s metabolism is an inter-connected network of enzymes, wherein the flux through an enzyme A might depend on the flux through an enzyme B providing substrate for the enzyme A. To account for this intracellular coupling of cellular metabolic processes, the transcriptomic and metabolomic data were linked with a metabolic network model to facilitate the description and understanding of changes in metabolic adaptations and responses [22]. Briefly, previously described methods [13–16] were used to integrate time-resolved transcriptomic and metabolomic data obtained from KAE609-treated or PA21A092-treated Dd2^A211V^ with a genome-scale metabolic network model of *P. falciparum*. The time-dependent data obtained under each condition serve as constraints in the metabolic network modelling and allow for simulating and representing the complex coupled nature of the altered metabolic states.

In concordance with the experimental culture conditions, it was assumed that both compounds resulted in approximately 50% reduction in the biomass of the treated parasites as compared to the untreated parasite. The built-in MATLAB function *trapz* was used to numerically integrate the simulated rates of essential macromolecules and metabolites in untreated and treated parasites (Table 3). Hierarchical clustering analysis on model-predicted metabolism of malaria parasites used the built-in MATLAB function *clustergram*, implementing the Ward’s algorithm and the Euclidean distance metric to group metabolic fluxes with similar temporal profiles between the untreated wildtype and the treated mutants.

## Results

### Concentration-dependent effects of PA21A092 and KAE609 on Dd2 wildtype and mutant morphologies

**Fig. 1:**
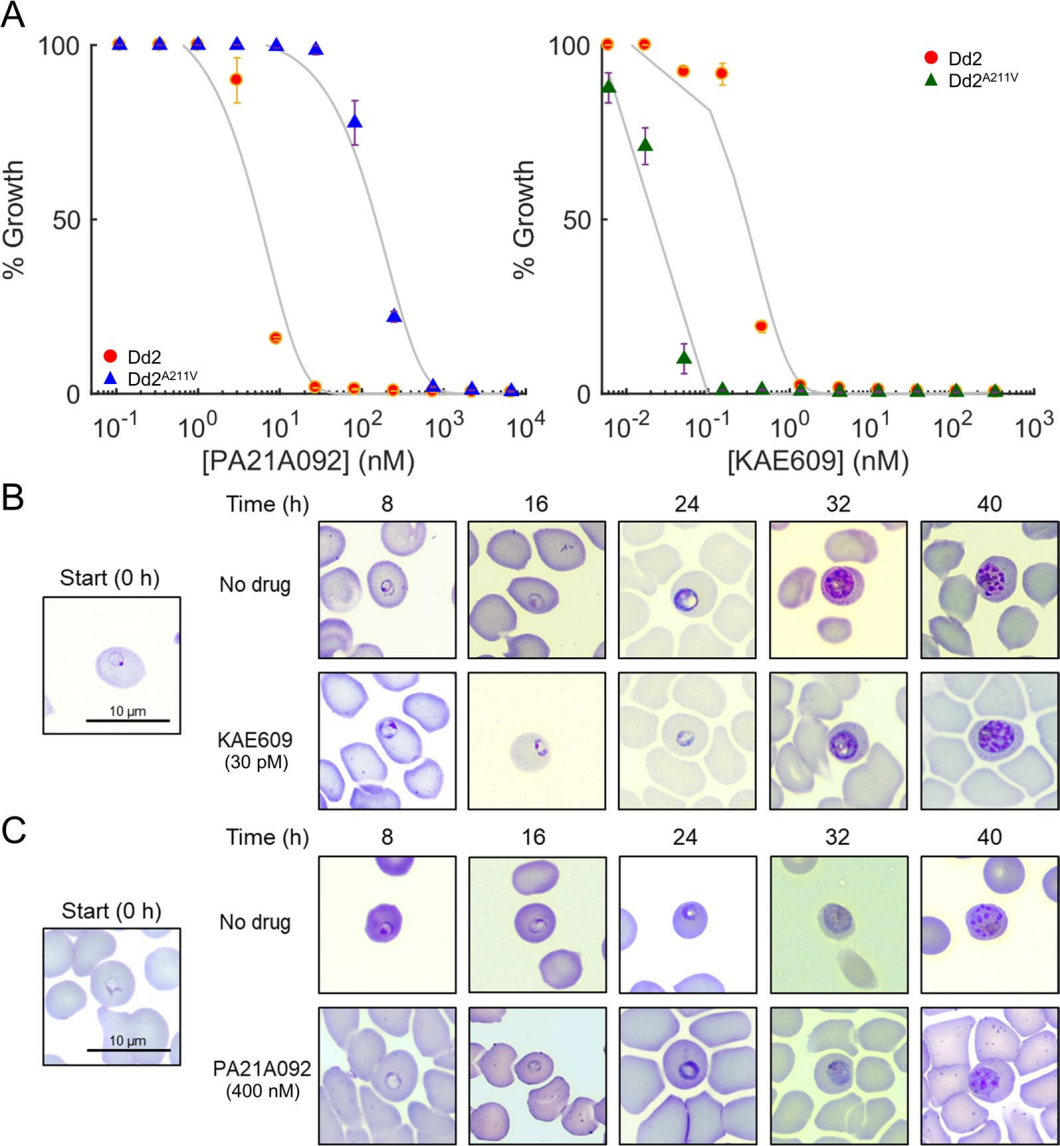
Effects of KAE609 and PA21A092 on the parasite-infected erythrocyte cultures. **A** Growth inhibition curves of Dd2 infected and Dd2^A211V^ infected erythrocyte cultures by PA21A092 and KAE609 compounds. The error bars show one standard deviation from an average of three technical measurements. The grey lines denote least-square fits to the experimental data. **B** Giemsa-stained morphologies of Dd2^A211V^ infected erythrocytes in the absence and presence of a sublethal dose (30 pM) of KAE609. **C** Giemsa-stained morphologies of Dd2^A211V^ infected erythrocytes in the absence and presence of a sublethal dose (400 nM) of PA21A092

In this study, the metabolic responses of the *P. falciparum* parasites exhibiting decreased PA21A092 sensitivity and increased KAE609 sensitivity due to mutations in the PfATP4 protein were characterized. The Dd2^A211V^ strain was used because the A211V mutation was generated through genome editing and can be compared to isogenic parental Dd2 parasites. Figure 1A shows dose–response curves of the Dd2^A211V^ and the parental Dd2 strains, demonstrating their decreased and increased susceptibility to PA21A092 and KAE609, respectively, resulting from the A211V mutation in the PfATP4 protein. Next, concentrations of PA21A092 and KAE609 were identified that inhibited the ability of synchronized ring stage parasites to replicate 40 h post treatment. Parasitaemia at the 70-h time point was measured to ensure that any parasite with the ability to replicate had done so. The sublethal (30–70% inhibition) doses were examined to capture adaptations associated with the treatment instead of downstream changes associated with parasite death. Additional file 1: Fig. S1 shows the effects of different concentrations of PA21A092 and KAE609 on synchronized cultures of Dd2^A211V^-infected erythrocytes. These experiments suggested that sublethal doses of 400 nM PA21A092 or 30 pM KAE609 would be appropriate to cause 30–70% reduction in replication of Dd2^A211V^ parasites.

To ascertain the effects of the identified sublethal PA21A092 and KAE609 doses on parasite morphologies, Giemsa-stained slides of infected erythrocyte cultures were analysed at 0-, 8-, 16-, 24-, 32-, and 40 h of the experiment. Figure 1B, C compare morphologies of KAE609-treated and PA21A092-treated Dd2^A211V^ cultures with that of untreated Dd2^A211V^. There appears to be a minor, but statistically significant (p < 0.0001), influence of PA21A092 on the number of merozoites per schizont (40 h, Fig. 1C). The merozoite number per schizont remained unchanged in the KAE609-treated Dd2^A211V^ (40 h, Fig. 1B) (Additional file 2: Fig. S2). To quantify the effect of the sublethal doses, i.e., 400 nM PA21A092 and 30 pM KAE609, on Dd2^A211V^ parasitaemia, we again assessed the reduction in parasite replication (relative to a parallel untreated culture) at the 70 h time point of the experiment and found that the identified doses resulted in approximately 45% and 70% inhibition of Dd2^A211V^ growth, respectively (Additional file 3: Fig. S3). Once it was confirmed that both compounds resulted in the desired stress on parasite replication and morphologies, samples were sent to collect transcriptomic and metabolomic data of the treated cultures during one complete IDC.

### Transcriptomic alterations in PA21A092-treated and KAE609-treated mutants

**Fig. 2:**
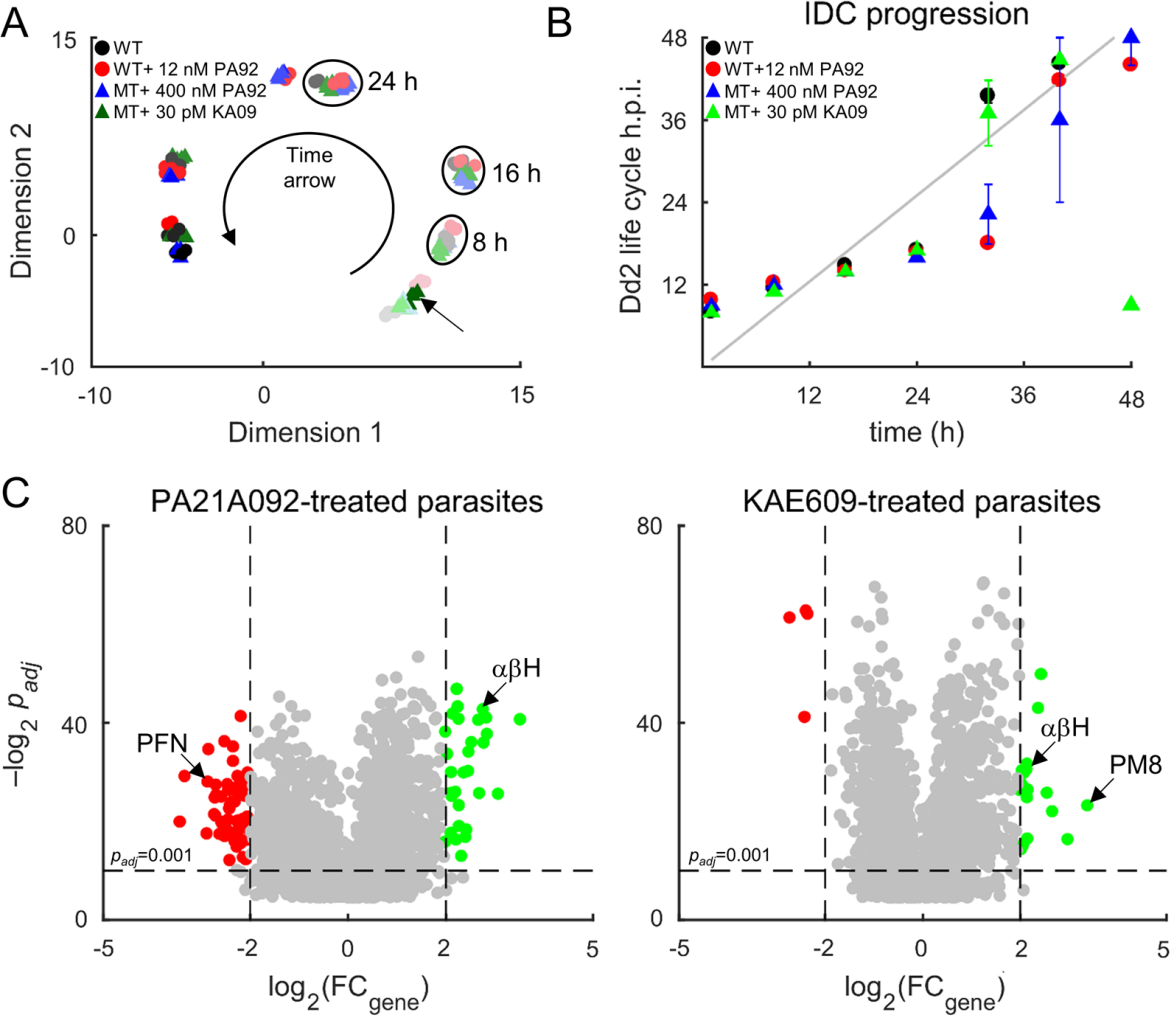
Transcriptomic profile of KAE609-treated and PA21A092-treated *P. falciparum*. **A** Dimensionally reduced transcriptomic data from untreated wildtypes (WT), PA21A092-treated wildtypes (WT + 12 nM PA92), PA21A092-treated mutants (MT + 400 nM PA92), and KAE609-treated mutants (MT + 30 pM KA09). Markers with lightest shade of a colour denotes 0 h time point, while the darkest shade of a colour denotes 48 h time point. Irrespective of the treatment compound, data from each time point group together (ellipses), except for the 48 h data from KAE609-treated mutants (arrow). **B** Time occurrence of the maximum Pearson correlation coefficient across the intraerythrocytic developmental cycle (IDC) of the hourly sampled data of Llinas et al. [20] (ordinate) with the culture systems studied here (abscissa). If the untreated and the treated parasites in the culture systems studied here progressed at a rate identical to the experiments of Llinas et al. [20], then the black, red, blue, and green markers would appear on top of each other along the grey line. It was found that the sublethal doses of 400 nM PA21A092 and 30 pM KAE609, respectively, caused minor perturbations to the IDC progression of the wildtypes (red circle) and the mutants (blue triangle, green triangle). **C** Significantly altered gene abundances in PA21A092-treated and KAE609-treated mutant parasites. The green markers denote log_2_ (FC_gene_) >  + 2 and red markers represent log_2_ (FC_gene_) < − 2. Here, FC_gene_ denotes fold change in average expression of a gene due to the treatment relative to its average expression in the parental Dd2 parasite. The average is based on 1000 bootstrap calculations (see Methods). αβH, alpha–beta hydrolase; h.p.i., hours post infection; KA09, KAE609 compound; PA92, PA21A092 compound; *PFN* profiling, *PM8* plasmepsin VIII

For identification of any major alterations in gene expression profiles of PA21A092-treated and KAE609-treated mutant parasites, a dimension-reduction method (t-SNE) was employed that included transcriptomic data from untreated parental Dd2 and PA21A092-treated Dd2 [16]. Figure 2A shows the time-resolved transcriptomic data, along the two t-SNE dimensions, from untreated Dd2, PA21A092-treated Dd2, PA21A092-treated mutant Dd2^A211V^, and KAE609-treated mutant Dd2^A211V^. With the use of the dimension-reduction technique, it was found that transcriptomic data from the parental and mutant strains at the sampled time points, regardless of culture conditions (untreated or drug-treated), grouped together, except for the 48-h transcriptomic data from KAE609-treated mutants (arrow, Fig. 2A).

For quantification of the effects of PfATP4 inhibitors on IDC progression, the time-resolved transcriptomic data from the PA21A092-treated and KAE609-treated mutant parasites were compared with an earlier hourly sampled transcriptomic dataset [20]. The transcriptomic data from untreated parental Dd2 [16] and PA21A092-treated Dd2 [16] were also included as positive controls of normal IDC progression and the effect of a PfATP4 inhibitor on the IDC progression. The Pearson correlation coefficient was calculated between each one of our collected datasets with the earlier hourly sampled Dd2 transcriptomic data to identify where the maximum correlation occurs for each of our sampled time points. Figure 2B shows the time point of maximum correlation between the datasets of this study and the hourly sampled data. If the data from this study had a perfect correspondence with the literature data, all markers would lie on the diagonal in the figure, whereas a marker above (below) the diagonal line indicates that the historical IDC data occurs later (earlier) than in the culture system used in this study. These results suggest that 48-h transcriptomic data from KAE609-treated mutants are akin to 8-h transcriptomic data from the literature data [20], and 0-h transcriptomic data from untreated and the treated parasites (Fig. 2B). During the blood stage, the malaria parasites are locked into a rigid, cascade-like transcriptional program repeating expression of specific genes at specific point of time in their 48-h cycle [23]. Although the KAE609-treated parasites maintain their transcriptional program for the majority of the sampled time points, they deviate substantially from their rigid transcriptional program at the 48-h time point.

For identification of significantly altered gene abundances due to the sublethal dose treatment, a two-way ANOVA was used and the average fold change in abundance of each gene (FC_gene_) due to the treatment relative to that in the untreated parental line was computed. Figure 2C shows significantly altered genes using green (log_2_FC_gene_ > +2*p*_adj_ < 0.001) and red (log_2_FC_gene_ < -2 *p*_adj_ < 0.001). It was found that most of the significantly altered genes (~ 90%) encode non-coding RNAs, exported proteins, or proteins of unknown function (Additional file 7: Table S2). In a prior work [14], it was found that expression of these genes tends to vary substantially between independent experiments. Among the significantly altered genes, only a few genes were found that appeared to be specifically related to the two treatment conditions, e.g., profilin (PFN downregulation for PA21A092, Fig. 2C) and alpha–beta hydrolase (αβH upregulation for both PA21A092 and KAE609, Fig. 2C). The αβH enzyme has hydrolase activity and utilizes adenosine triphosphate (ATP), or guanosine triphosphate (GTP), as substrate. An increased expression of αβH suggests a compensatory role for this enzyme in the KAE609-treated and PA21A092-treated parasites.

Figure 2C also shows statistically significant upregulation of the gene encoding plasmepsin VIII (PM8) in the KAE609-treated parasites. PM8 belongs to a group of diverse aspartic proteases called plasmepsins and is essential during the mosquito stage [24], but is potentially dispensable, based on the piggyBac screen [25], during the blood stage. Interestingly, there was significant upregulation of the gene encoding PM8 in the PA21A092-treated wildtypes parasites (Table S1 of [16]), but not in the PA21A092-treated mutants. Because the parental Dd2 strain is sensitive to PA21A092, while the Dd2^A211V^ strain is resistant and hypersensitive to PA21A092 and KAE609, respectively, these results suggest a role for PM8 under drug-treated conditions that remains to be elucidated.

For identification of functional implications of altered gene abundances in *P. falciparum*, an enrichment analysis was performed using gene sets available in the Malaria Parasite Metabolic Pathway (MPMP) database [26]. Table 1 lists gene sets that were altered in KAE609- and PA21A092-treated Dd2^A211V^ mutants but unaltered in three other datasets collected using identical methods, where the culture medium only differed in the perturbations, i.e., hypoxanthine deprivation [14], apicoplast disruption [17], and fosmidomycin treatment [15] in the NF54 strain. The same gene sets were found to be altered in KAE609- and PA21A092-treated mutants as in the PA21A092-treated Dd2 strain. Thus, the identified gene sets appear to be associated with the disruption of PfATP4-regulated Na^+^ homeostasis per se, rather than the sensitivity of either KAE609 or PA21A092.

**Table 1:** Gene sets altered in KAE609-treated and PA21A092-treated mutant parasites

Gene set description	N_gene_	Percentage of altered genes (%)					
		Dd2^A211V^		Dd2	NF54		
		KA09^a^	PA92^a^	PA92^b^	HXN^c^	API^d^	FOS^e^
Respiratory chain proteins	48	18.8	22.9	8.3	0.0	0.0	2.1
Mitochondrion electron flow	37	16.2	18.9	10.8	2.7	0.0	0.0
Biogenesis of cytochrome oxidase	29	13.8	6.9	6.9	3.4	0.0	3.4
Copper transport	21	28.6	14.3	14.3	4.8	0.0	0.0
Centriole proteins	21	19.0	23.8	14.3	0.0	0.0	4.8
Cytochrome C biogenesis	18	22.2	22.2	11.1	5.6	0.0	0.0
Mitochondrial disulphide relay system	13	15.4	7.7	15.4	7.7	0.0	0.0
Lysine methylation and demethylation	11	18.2	18.2	9.1	0.0	0.0	0.0

*KA09* KAE609-treated mutants, *PA92* PA21A092-treated mutants

^a^This work

^b^PA92, PA21A092-treated Dd2 [16]

^c^HXN, hypoxanthine-limited NF54 [14]

^d^API, apicoplast-disrupted NF54 [17]

^e^FOS, fosmidomycin-treated NF54 [15]

### Metabolomic alterations in PA21A092-treated and KAE609-treated mutant parasites

**Fig. 3:**
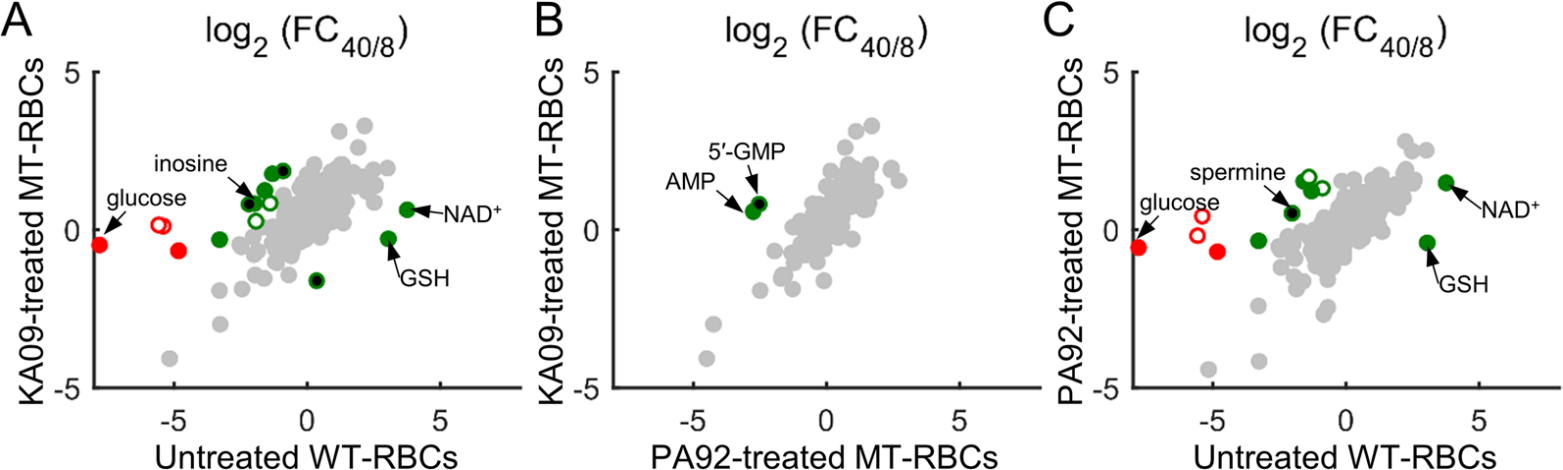
Substantially altered metabolite abundances in KAE609-treated and PA21A092-treated mutant parasite cultures. **A** FC_40/8_ values of metabolites detected in KAE609-treated cultures of mutant parasites compared with untreated cultures of wildtype parasites. **B** FC_40/8_ values of metabolites detected in KAE609-treated cultures of mutant parasites compared with PA21A092-treated cultures of mutant parasites. **C** FC_40/8_ values of metabolites detected in PA21A092-treated cultures of mutant parasites compared with untreated cultures of wildtype parasites. Here, FC_40/8_ denotes average fold change in abundance of a metabolite at the 40 h time point relative to its abundance at the 8 h time point. The average is based on 10,000 bootstrap calculations (see Methods). Red markers denote metabolites having fourfold different FC_40/8_ after treatment as compared to untreated conditions, while grey markers denote metabolites having less than twofold difference; all other metabolites are shown using green circles. Open red and green markers denote metabolites that tend to vary substantially over time in uninfected erythrocytes [49], while markers that have been filled black denote metabolites with no variability information. *5′-GMP* 5′ guanosine monophosphate, *AMP* adenosine monophosphate, *GSH* reduced glutathione, *KA09* KAE609 or cipargamin, *MT-RBC* erythrocytes infected with mutant parasites, *NAD*^*+*^ nicotinamide adenine dinucleotide, *PA92* pyrazoleamide compound PA21A092, *WT-RBC* erythrocytes infected with wildtype parasites

To identify treatment-specific alterations in metabolite abundances over time, the FC_40/8_ values of metabolites that are common to all three cultures, i.e., untreated PfDd2 and KAE609- and PA21A092-treated Dd2^A211V^ mutant cultures, were computed. Because the ring-stage parasites are metabolically less active than late-stage parasites, a comparison of FC_40/8_ values between untreated and the treated condition should capture the effects of the sublethal dose on metabolic progression during the IDC. Figure 3A–C shows the pairwise comparison of the estimated FC_40/8_ values between the three cultures. It was found that KAE609 and PA21A092 induced almost identical alterations in metabolite abundances in Dd2^A211V^ cultures as compared to the Dd2 cultures (Fig. 3A, C). In fact, only a few metabolites, such as inosine (Fig. 3A), adenosine monophosphate (AMP, Fig. 3B), 5′ guanosine monophosphate (5′-GMP, Fig. 3B), and spermine (Fig. 3C), appeared substantially different between the KAE609- and PA21A092-treated cultures, suggesting differences in purine and polyamine metabolism of these two cultures.

For quantification of temporal alterations in metabolite abundances, the average fold change in abundance of each metabolite relative to its abundance at 0 h (FC_0h_) of metabolites common to both KAE609-treated and PA21A092-treated mutant cultures was computed. Table 2 lists metabolites having substantially (varying by more than twofold, |∆log_2_|> 1) different FC_0h_ values between the culture conditions.

**Table 2:** Substantially altered metabolites in KAE609-treated and PA21A092-treated cultures

Pathway	Subpathway	Metabolite	log_2_ (FC_0h_)^b^	
			+ PA92	+ KA09
Amino Acid	Arginine and proline metabolism	2-oxoarginine^a^	1.15	− 0.54
		Arginine	0.75	− 1.20
		N-acetylarginine	1.30	− 0.44
	Lysine metabolism	Fructosyllysine	0.79	− 1.25
	Methionine, cysteine, and taurine metabolism	Cysteine	1.46	− 0.90
	Tyrosine Metabolism	3-(4-hydroxyphenyl)lactate	− 0.41	− 1.89
Carbohydrate	Pentose phosphate pathway	Sedoheptulose-7-phosphate	1.47	3.39
Cofactors and Vitamins	Biotin metabolism	Biotin	1.05	− 0.50
Energy	Tricarboxylic acid cycle	Aconitate	1.18	− 0.60
		Citrate	0.87	− 0.75
Lipid	Fatty acid metabolism	2,4-dihydroxybutyrate	− 2.25	− 0.74
	Bile acid metabolism	Glycochenodeoxycholate	1.33	-0.20
		Glycocholate	1.56	− 0.36
		Glycodeoxycholate	1.52	− 0.27
Nucleotide	Purine metabolism	Inosine	− 0.41	1.62

*KA09* KAE609-treated erythrocyte cultures infected with mutant parasites, *PA92* PA21A092-treated erythrocyte cultures infected with mutant parasites

^a^Metabolite identified based on m/z ratio alone with no external standard for validation

^b^FC_0h_ denotes average increase (or decrease) in abundance of a metabolite over time relative to its abundance at 0 h of the experiment

It was found that bile acid metabolites accumulated in PA21A092-treated mutants but decreased in the KAE609-treated mutants (see Table 2). In the liver and intestine, Na^+^-dependent transporters support absorption and secretion of bile acids [27]. Because both PA21A092 and KAE609 are Na^+^ homeostasis disruptors, these results indicate that Na^+^ homeostasis is differentially altered in PA21A092-treated and KAE609-treated mutant parasites, but whether human erythrocytes or *Plasmodium* parasites encode bile acid transporters is unknown.

### Metabolism of PA21A092-treated and KAE609-treated parasites

**Fig. 4:**
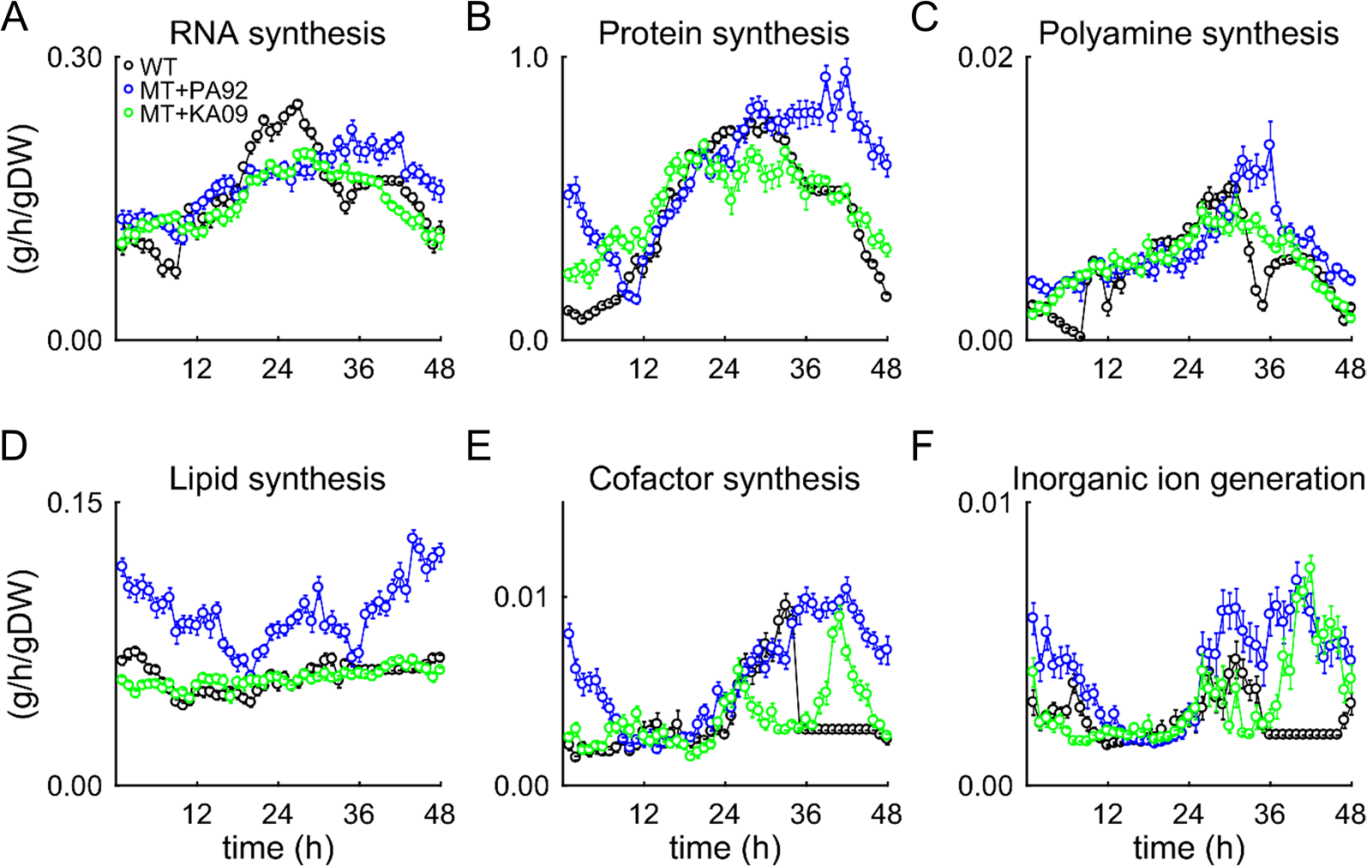
Model-predicted rates of macromolecular syntheses in KAE609-treated and PA21A092-treated mutant parasites. **A** RNA synthesis. **B** Protein synthesis. **C** Polyamine synthesis. **D** Lipid synthesis. **E** Cofactor synthesis. **F** Inorganic ion generation. The error bars indicate one standard deviation (SD) of 20 simulations computed after adding random Gaussian noise with zero mean and a SD based on the respective transcriptomic data. g/h/gDW, grams per hour per gram dry weight of the parasite; MT + KA09, KAE609-treated *P. falciparum* Dd2^A211V^; MT + PA92, PA21A092-treated *P. falciparum* Dd2^A211V^; *WT* untreated *P. falciparum* Dd2

For delineation of treatment-induced metabolic alterations, the time-resolved data were integrated with a genome-scale metabolic network model of the parasite [14–16]. This model has been successfully used to predict the effects of hypoxanthine deprivation [14] and fosmidomycin treatment [15] on NF54 strain parasites, and PA21A092 treatment [16] on Dd2 parasites. Figure 4 shows model-predicted rates of macromolecule syntheses in untreated Dd2, PA21A092-treated Dd2^A211V^, and KAE609-treated Dd2^A211V^ parasites. These results suggest that the rates of macromolecule synthesis of the wildtype and mutant parasites between the three conditions are largely comparable, except for the rate of lipid synthesis. Lipid synthesis was substantially higher in the PA21A092-treated mutants as compared to the wildtype Dd2 and KAE609-treated mutants (Fig. 4D).

For quantification of the net accumulation or reduction of essential macromolecules and metabolites, the model-predicted rates of synthesis in the presence of the two drugs were numerically integrated. Table 3 lists the fold change in net synthesized amount (Δm) of essential macromolecules and metabolites during the IDC in the presence of either compound. PA21A092-treated mutants produced ~ 60% more phospholipids than the untreated parasites, whereas phospholipid production in the KAE609-treated mutants was unchanged during the IDC. In contrast to a recent study [16] that reported an increase in myoinositol synthesis rate during late stages of PA21A092-treated wildtypes, this study found enhanced myoinositol synthesis during the entire IDC of PA21A092-treated mutants (Additional file 4: Fig. S4). Because the Dd2^A211V^ strain is resistant to PA21A092 and phosphatidylinositol 3-phosphate (downstream product of myoinositol) is associated with drug resistance in *P. falciparum* [28], these results also suggest a role for phospholipid signalling in PA21A092 resistance.

**Table 3:** Macromolecules and metabolites accumulated in KAE609- and PA21A092-treated parasites

Macromolecule	$\Delta {\text{m}}{{^a}} = \frac{\int {\text{v}}_{\text{PA92}}^{\text{t}}}{\int {\text{v}}_{\text{CTL}}^{\text{t}}}$	$\Delta {\text{m}}{{^a}}\text{ } = \frac{\int {\text{v}}_{\text{KA09}}^{\text{t}}}{\int {\text{v}}_{\text{CTL}}^{\text{t}}}$
DNA	1.10	1.19
RNA	1.07	0.94
Protein	1.30	1.06
Phospholipid	1.59	0.99

Metabolite	$\Delta {\text{m}}$	$\Delta {\text{m}}$
Ammonium	3.25	1.76
S-adenosyl-L-methionine	2.56	1.29
Nicotinamide-adenine-dinucleotide	1.88	1.04
Protoheme	1.83	0.50
10-formyltetrahydrofolate	1.66	0.98
5–10-methylenetetrahydrofolate	1.44	0.87
Pyridoxal-5-phosphate	1.32	1.15
Coenzyme-A	1.31	1.00
Putrescine	1.26	1.27
Spermidine	1.25	0.79

^a^fold change in net synthesized amount of a macromolecule (or metabolite). Here, $\int {\text{v}}_{\text{X}}^{\text{t}}$ denotes total accumulated amount of a macromolecule (or metabolite), computed numerically by integrating its synthesis rate (v) over the 48 h intraerythrocytic developmental cycle. Where, X is equal to untreated (CTL), KAE609-treated (KA09), or PA21A092 treated (PA92) conditions

**Fig. 5:**
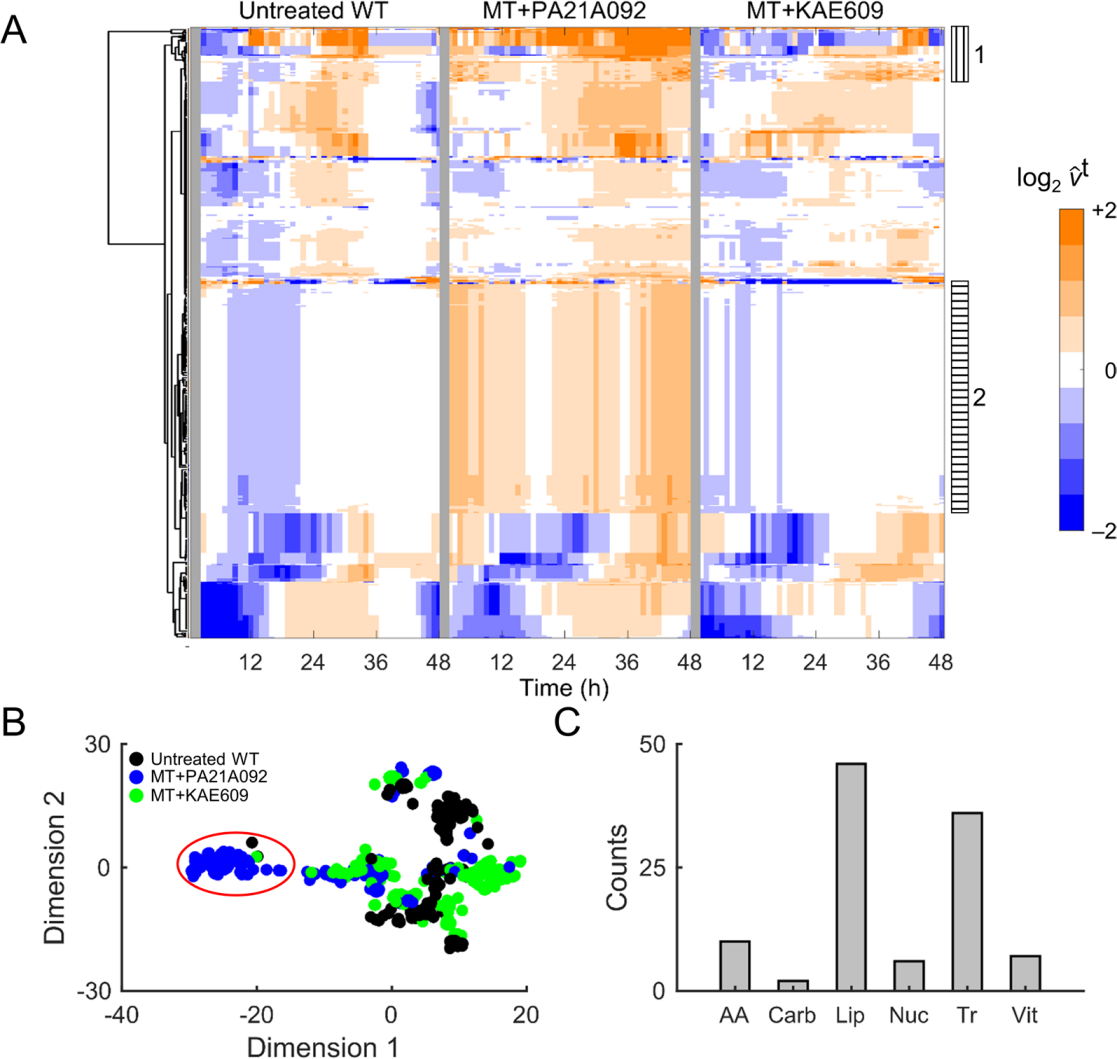
Model-predicted metabolism of untreated Dd2, PA21A092-treated Dd2^A211V^, and KAE609-treated Dd2^A211V^. **A **Hierarchical clustering analysis of non-zero metabolic fluxes in untreated wildtypes (WT) and mutants treated with pyrazoleamide compound PA21A092 (MT + PA21A092) and spiroindolone compound KAE609 (MT + KAE609). The cluster analysis revealed metabolic reactions having substantially different flux in the presence of PA21A092, but not in the absence of drugs or in the presence of KAE609, annotated using 1 (vertical stripes) and 2 (horizontal stripes).${\widehat{\text{v}}}^{\text{t}}$ denotes temporal flux of an enzyme after normalization by its median value in the untreated WT parasites. B) Metabolic fluxes in wildtypes (WT; black), PA21A092-treated mutants (MT; blue), and KAE609-treated mutants (MT; green) along two reduced dimensions. The red ellipse contains metabolic reactions, primarily, from PA21A092-treated mutants. **C** Metabolic pathways associated with the metabolic reactions contained in the red ellipse (**B**), *Cluster* 1 (**A**), and *Cluster 2* (**A**). *AA* amino acid reactions, *Carb* carbohydrate reactions, *Lip* lipid reactions, *Nuc* nucleotides, *Tr* transport reactions; *Vit* cofactors and vitamin reactions

For identification of similarities and dissimilarities in the metabolism of PA21A092- and KAE609-treated mutants, hierarchical clustering analysis was performed on model-predicted metabolism of untreated wildtype Dd2 and the treated mutants. Figure 5A illustrates non-zero enzymatic fluxes in the untreated wildtype Dd2 and the treated mutants. The cluster analysis revealed two clusters (annotated 1 and 2, Fig. 5A) containing enzymes having substantially higher flux in the PA21A092-treated mutants as compared to the untreated wildtype Dd2 and KAE609-treated mutants.

t-SNE and two-way ANOVA were then employed to identify significantly (*p* < 0.001) altered metabolic reactions in the PA21A092-treated mutants as compared to the KAE609-treated mutants. Figure 5B shows metabolic reactions of the untreated wildtype Dd2 and the treated mutants along the two t-SNE dimensions, revealing a cluster (red ellipse, Fig. 5B) containing metabolic reactions primarily from the PA21A092-treated parasites. Figure 5C characterizes the metabolic reactions circumscribed in Fig. 5B and contained in *Cluster* 1 and 2 (Fig. 5A) into six metabolic pathways, indicating that most (~ 43%) of the significantly altered reactions belonged to lipid metabolism. In addition to lipid metabolism, these reactions belong to purine metabolism, membrane transport, methylation cycle, and fatty acid metabolism (Additional file 8: Table S3). It was found that these diverse pathways provide precursors necessary for synthesis of phospholipids, i.e., phosphatidylcholine, phosphatidylserine, phosphatidylethanolamine, and phosphatidylinositol.

**Fig. 6:**
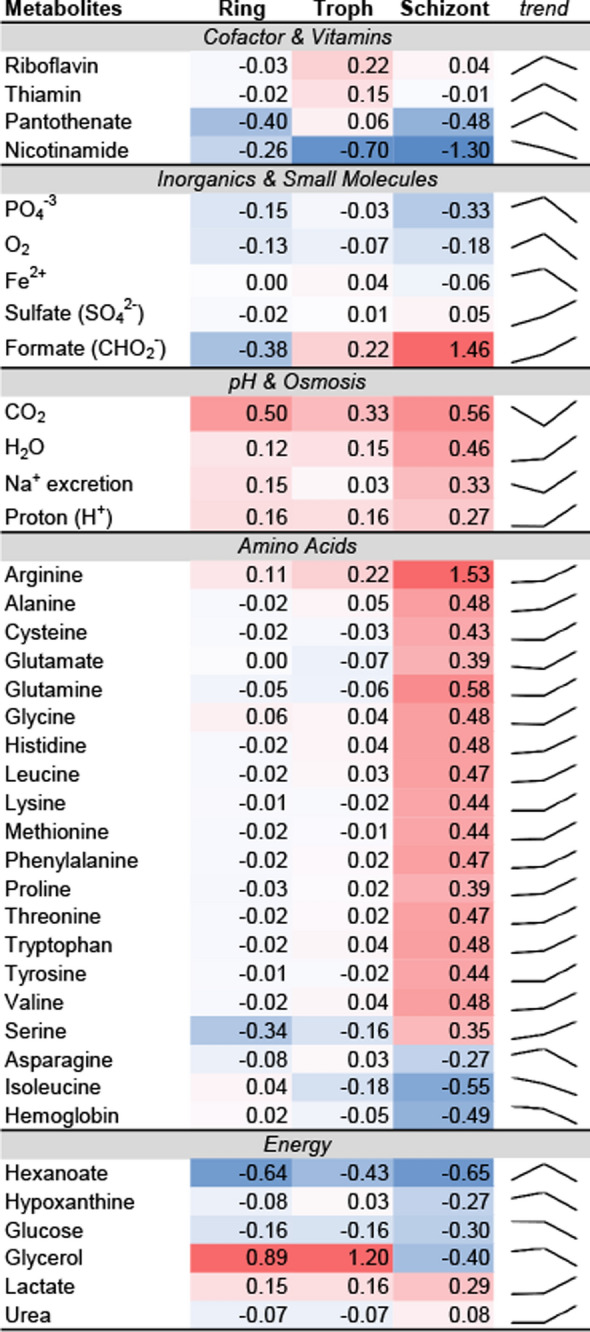
Average difference of model-predicted fluxes transporting the listed metabolites in PA21A092-treated and KAE609-treated mutants. The flux difference was averaged to capture ring (1–18 h), trophozoite (19–32 h), and schizont (33–48 h) stages of blood-stage malaria parasites. Prior to taking the average of difference, the flux-difference value of a metabolite was normalized by its median value in the Dd2 wildtype. A negative (or positive) value, under the ring, trophozoite (troph), or schizont column, suggests that the metabolic flux is more negative (or positive) in PA21A092-treated mutants as compared to the KAE609-treated mutants. The *trend* lines capture gross difference in metabolic fluxes during ring, trophozoite, and schizont stages of the two mutants

Within the metabolic network model, exchange fluxes of the PA21A092-treated and KAE609-treated mutants captured nutrient uptake alterations associated with the resistance and hypersensitivity of the two drugs. Therefore, the difference between model-predicted exchange fluxes of PA21A092-treated and KAE609-treated mutants was computed to quantify differences in nutrient requirements of the two cultures. Figure 6 shows exchange-flux differences between the two cultures averaged to capture ring, trophozoite, and schizont stages of blood-stage parasites. PA21A092-treated Dd2^A211V^ maintained energy generation, haemoglobin degradation, isoleucine uptake, Na^+^ excretion, and osmosis better than the KAE609-treated Dd2^A211V^. These results suggest that PA21A092-treated mutants utilize arginine, formate, and nicotinamide more efficiently than the KAE609-treated mutants. A closer examination into these metabolites indicated an increased pyrophosphate generation via nicotinate phosphoribosyltransferase in the PA21A092-treated mutants but not in the KAE609-treated mutants. *Plasmodium falciparum* encodes two proton pyrophosphatases: (*i*) potassium-dependent (PF3D7_1456800) and (*ii*) potassium-independent (PF3D7_1235200), catalysing pyrophosphate to fuel their proton pumping activity. An increased expression of PF3D7_1456800 was found at all time points (except 40 h and 48 h, Additional file 5: Fig. S5), and an increased expression of PF3D7_1235200 was found at the 40 h and 48 h time points in the PA21A092-treated mutants. The malaria parasite predominantly uses a V-type H^+^ ATPase to regulate its intracellular pH [29], which is disrupted after treatment with various PfATP4 inhibitors [30]. Therefore, these results suggest a role for these pyrophosphatases in maintenance of intracellular pH to compensate for the inhibition of PfATP4 in the treated parasites.

## Discussion

Spiroindolones and pyrazoleamides belong to a novel class of anti-malarial drugs that work by perturbing Na^+^ homeostasis in *P. falciparum*. The PfATP4 protein maintains the Na^+^ gradient across the parasite plasma membrane [31] and point mutations in PfATP4 modulate parasites’ sensitivity to PfATP4-targeting drugs, such as spiroindolones and pyrazoleamides [11]. In this work, an isogenic pair of Dd2 parasite lines containing wildtype or the A211V mutation in PfATP4 were used to investigate metabolic alterations associated with resistance against PA21A092 (a pyrazoleamide compound) and increased sensitivity to KAE609 (a spiroindolone compound). For identification of PfATP4-related mechanisms that regulate drug sensitivity in *P. falciparum*, sublethal doses of PA21A092 (and KAE609) that did not cause substantial changes to parasite morphology until the 40-h time point of the experiment, but still caused approximately 30–70% inhibition of Dd2^A211V^ replication during the second IDC, were determined. At the determined sublethal concentration of the two compounds, time-resolved metabolomic and transcriptomic data were collected from Dd2^A211V^ cultures during the IDC, and a series of quantitative and metabolic data analyses were performed.

### Transcriptomic and metabolomic alterations in the treated Dd2^A211V^

As compared to the PA21A092-treated mutants, KAE609 treatment caused substantially less alteration in the transcriptional program of the parasite during the IDC. In fact, the number of significantly altered genes in the KAE609-treated parasites was approximately 20% of that in the PA21A092-treated parasites. Consequently, the number of genes altered under both conditions was also low (only 10 genes were altered due to both PA21A092 and KAE609). Of the 10 genes, one gene (PF3D7_0823400) encoded *P. falciparum* αβH and was significantly upregulated (> twofold; *p*_adj_ < 0.001) under both the conditions. It was found that the expression of this gene was downregulated in response to treatment by other anti-malarials, such as artemisinin [32], atovaquone [32], chloroquine [32], and tafenoquine [32]. Because αβH can hydrolyse ATP, these results suggest a compensatory role for this protein in the PA21A092-treated and KAE609-treated mutants. Most of the other nine genes encode RIFIN proteins or non-coding RNAs, which based on a prior work [14] tend to vary substantially, yet non-specifically, between different perturbations.

A recent effort [16] found that PA21A092 treatment causes a substantial increase in the synthesis of myoinositol, a precursor of phosphatidylinositol-4,5-bisphosphate (PIP_2_), in late-stage wildtype parasites. This study found significant downregulation (> twofold; *p*_adj_ < 0.001) of the gene (PF3D7_0932200) encoding profilin, a PIP_2_-binding protein, in the PA21A092-treated mutants, but not in the KAE609-treated mutants. The profilin protein modulates the activity of phosphoinositide 3-kinases (PI3Ks) by interacting with PIP_2_ and, thus, can have a profound effect on multiple signalling pathways, such as motility, proliferation, and apoptosis [33]. Under in vitro conditions [33], downregulation in profilin protein is accompanied by increased abundance of PIP_2_, a downstream product of myoinositol biosynthesis. Moreover, an expected consequence of Na^+^ homeostasis disruptors is cell swelling [34], which leads to PI3K activation in many cell types [35]. The PI3K protein phosphorylates PIP_2_ to yield phosphatidylinositol (3, 4, 5)-triphosphate (PIP_3_), which is known to activate Akt in most cells [36] and support cell survival [37]. Because overexpression of *Plasmodium* Akt (PfAkt) leads to clinical-level artemisinin resistance in *P. falciparum* [28], it is likely that the PI3K/PfAkt pathway also plays a role in modulating sensitivities of pyrazoleamide and spiroindolone drugs in malaria parasites.

In contrast to the transcriptomic data analysis, metabolic alterations in the PA21A092-treated and KAE609-treated mutant cultures were mostly similar (Fig. 3A, C). An accumulation of purine metabolites (inosine, AMP, and GMP) was found in KAE609-treated mutant cultures as compared to the wildtype cultures and PA21A092-treated cultures, whereas an accumulation of a polyamine metabolite, spermine, was observed in the PA21A092-treated mutant cultures. At the outset, these results suggest only minor differences in the metabolism of the PA21A092-treated and KAE609-treated mutant parasites.

### Metabolic pathways associated with resistant and hypersensitive phenotypes

**Fig. 7:**
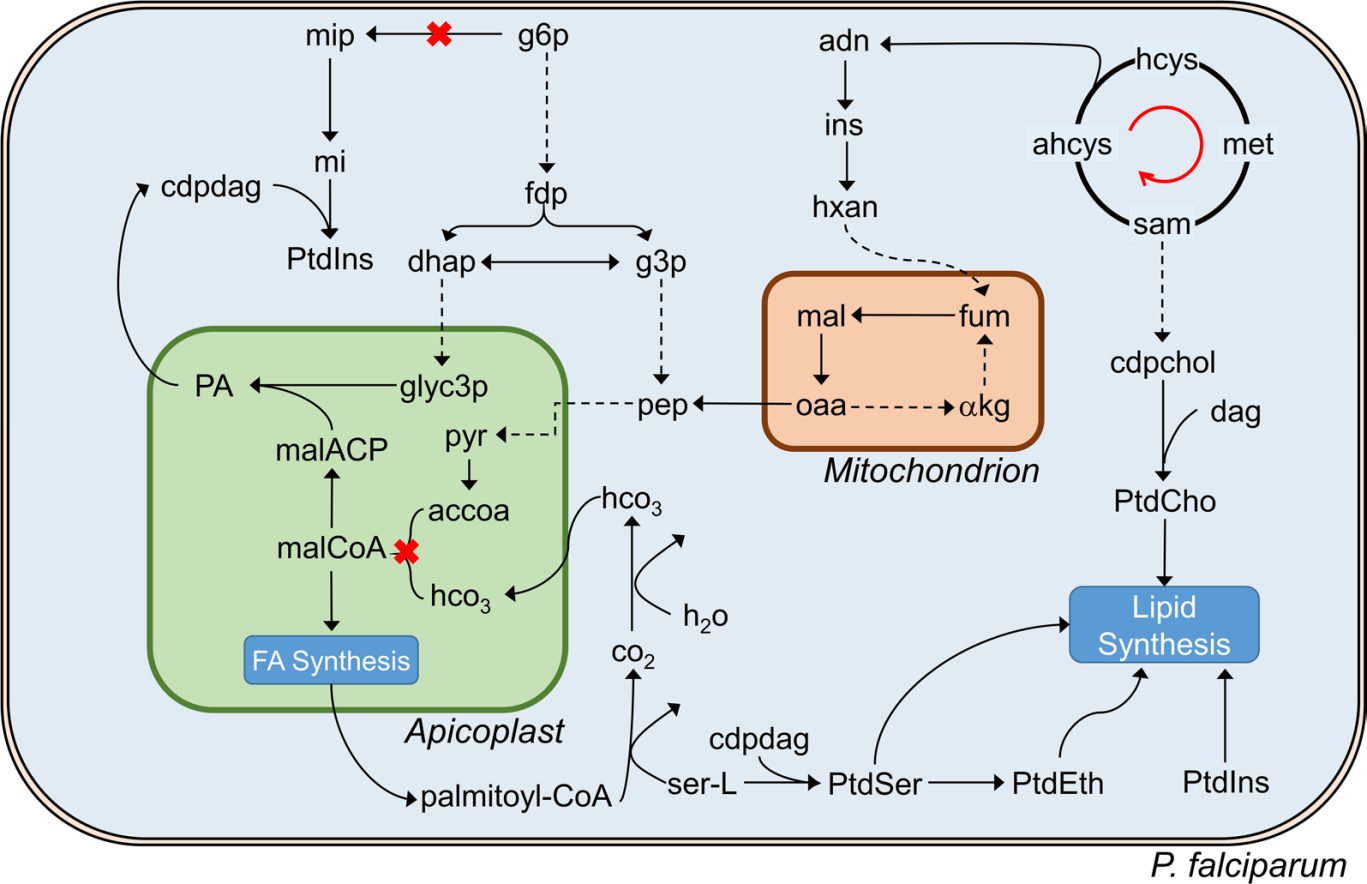
Metabolic reactions associated with the altered drug-sensitivity phenotypes of KAE609-treated and PA21A092-treated *P. falciparum* Dd2^A211V^ parasites (Fig. 5A). These reactions conduct de novo synthesis of phosphatidylinositol (PtdIns), phosphatidylserine (PtdSer), phosphatidylethanolamine (PtdEth), and phosphatidylcholine (PtdCho). The two red cross marks represent myoinositol phosphate synthase and acetyl-CoA (accoa) carboxylase enzymes, which are essential for de novo synthesis of myoinositol phosphate (mip) and malonyl CoA (malCoA), respectively. These precursors are essential for phosphatidylinositol and fatty acid synthesis in *P. falciparum*. The dashed lines indicate the presence of multiple enzymes, while solid lines indicate the presence of an individual enzyme. *αkg* α-ketoglutarate, *adn*, adenosine, *ahcys* S-adenosylhomocysteine, *cdpchol* cytidine diphosphocholine, *cdpdag* cytidine diphosphate diacylglycerol, *CoA* coenzyme A, *dag* diacylglycerol, *dhap* dihydroxyacetone phosphate, *FA* fatty acid; *fdp* fructose diphosphate, *fum* fumarate, *g3p* glyceraldehyde 3-phosphate, *g6p* glucose 6-phosphate, *glyc3p* glycerol 3-phosphate, *hco*_*3*_ bicarbonate, *hcys* homocysteine, *hxan* hypoxanthine, *ins* inosine, *mal* L-malate, *malACP* malonyl acyl carrier protein, *met* L-methionine, *mi* myoinositol, *oaa* oxaloacetate, *PA* phosphatidic acid, *pep* phosphoenolpyruvate, *pyr* pyruvate, *sam* S-adenosylmethionine, *ser-L* L-serine

To pinpoint differences between the observed resistant and hypersensitive phenotypes, the quantitative data analysis was augmented using a genome-scale metabolic network model to identify a set of reactions comprised of primarily lipid metabolism reactions (Fig. 5B) that were almost exclusively associated with the resistant phenotype. Figure 7 schematically summarizes the interaction network of the metabolic reactions contained within the identified cluster. A recent publication [16] posited that enhanced synthesis of phosphatidylinositol might underlie drug-resistance development in *P. falciparum*. In agreement with that recent work, in this study metabolic reactions synthesizing phosphatidylinositol, phosphatidylethanolamine, phosphatidylserine, and phosphatidylcholine were significantly upregulated in the Dd2^A211V^ cultures exhibiting PA21A092 resistance, but not in Dd2^A211V^ cultures showing KAE609 hypersensitivity.

Na^+^ homeostasis disruptor drugs induce cell swelling in *P. falciparum* [38]. A consequence of cell swelling in HepG2 cells is the activation of protein kinases, such as [39], which in turn is responsible for activation of acetyl-CoA carboxylase enzyme [35]. The acetyl-CoA carboxylase (ACC) enzyme synthesizes malonyl-CoA (malCoA in Fig. 7), a precursor for Type II fatty acid synthesis in *P. falciparum* [40]. This study found an increased ACC flux and, consequently, an increased fatty acid synthesis (FA Synthesis in Fig. 7) in PA21A092-treated mutants but not in the KAE609-treated mutants. A downstream consequence of increased ACC flux is enhanced synthesis of phosphatidylserine (PtdSer in Fig. 7), a critical modulator of protein kinase B (Akt)-mediated cell survival [41]. Therefore, these results suggest that enhanced phospholipid synthesis, observed in PA21A092-treated mutants, is a downstream consequence of the Na^+^ homeostasis disruptor to promote *Plasmodium* proliferation.

Given that both compounds target the same protein and that the sublethal concentrations were selected based on their ability to have the same disruption on the IDC progression, the possibility cannot be excluded that differences in their sensitivity are partially due to their off-target effects. Compounds that interact with ion-channel transporters can have physiological effects beyond their primary target. To identify potential off-target effects of the two compounds, the computational prediction tool ToxProfiler was used to scan for drug-protein interactions that are known to causally link to phenotype-level adverse effects [42]. It was found that KAE609 interacts significantly with two proteins: ATP binding cassette subfamily B member 11 (ABCB11) and Gamma-aminobutyric acid receptor subunit alpha-1 (GABRA1) (Additional file 9: Table S4: Sheet 1). In the *P. falciparum* genome [43], there are five orthologs of ABCB11 and no orthologs of GABRA1. Of the five ABCB11-related genes, one gene, PF3D7_1339900, encoding ABC transporter B family member 5 (ABCB5) protein was found to have substantially higher expression in the PA21A092-resistant cultures as compared to the KAE609-sensitive cultures of Dd2^A211V^ (Additional file 9: Table S4: Sheet 2). The ABCB5 protein is a generic transporter of small ions, sugars, and peptides [44] and has been associated with resistance against chemotherapeutics, such as doxorubicin [45] and 5-fluorouracil [46], presumably via a drug-efflux mechanism [45]. This suggests that KAE609 may have an enhanced efficacy against the Dd2^A211V^ strain due to its interaction with the ABCB5 protein, causing an enhanced intracellular accumulation of KAE609 as compared to PA21A092. These results provide a parallel explanation for the differential sensitivities of PA21A092 and KAE609 in Dd2^A211V^.

### Limitations of the current study

In this work, the effects of the A211V mutation on PfATP4 function were not studied. A recent study has shown that another mutation (G358S) can alter binding affinities of the cipargamin drug and sodium ions for the PfATP4 protein [47]. Therefore, it is possible that physiological changes related to sodium homeostasis may occur in untreated malaria parasites carrying the A211V mutation. Herein, with the help of the metabolic network model, the effect of any sodium homeostasis alteration on parasite metabolism would be accounted for by the sodium-dependent phosphate transporter [48]. Therefore, the used modelling frame work would indirectly capture the effects of any mutation-associated compensatory adaptations in the parasite metabolism by integrating time-resolved transcriptomic data collected during the IDC. Moreover, in this work, the physiological differences between the Dd2 wildtypes and Dd2^A211V^ parasites were not compared because our primary goal was to identify parasitic responses associated with differential sensitivities of KAE609 (cipargamin) and PA21A092 compounds in the Dd2^A211V^ parasites.

Lastly, to simulate the effect of KAE609 (or PA21A092) on the metabolism of Dd2^A211V^ parasites during the entire IDC, time-resolved transcriptomic and metabolomic data were coupled with a metabolic network model, which uses explicit gene-protein reaction rules to predict enzymatic activities in the Dd2^A211V^ parasites. Because the modelling approach does not explicitly model the interaction of KAE609 (or PA21A092) with the PfATP4 protein, the metabolic adaptations identified herein are not necessarily associated with the differential sensitivities of the two chemicals; instead, these model-predicted adaptations represent global changes induced by the two chemicals in parasite metabolism, which may include toxic and non-toxic effects of the treatment in the *P. falciparum*.

## Conclusions

PA21A092, a pyrazoleamide compound, belongs to a novel class of anti-malarials that perturb Na^+^ homeostasis in *P. falciparum*. Recent evidence suggests that specific mutations in the PfATP4 protein confer resistance against PA21A092, but at the same time enhance efficacy of another Na^+^ homeostasis disruptor, KAE609. Here, the metabolic and transcriptomic changes in blood-stage *P. falciparum* Dd2^A211V^ exhibiting differential sensitivities against KAE609 and PA21A092 were examined to characterize physiological processes associated with the different responses to these compounds.

Sublethal compound concentrations were selected to limit parasite death during the first IDC. Negligible morphological changes were observed, yet the identified concentrations substantially reduced the ability of the parasites to replicate beyond the first IDC. As expected, the time-dependent transcriptional response of the Dd2^A211V^ strain, in the presence sublethal doses of either compound, was largely similar to the wildtype Dd2 strain and commensurate with the parasites successfully compensating for the drug pressure by maintaining the overall schedule of completing the first IDC.

The sublethal concentration of either compound generates a time-dependent differential gene signal that is mainly (~ 90%) dominated by non-functional genes exhibiting a highly variable transcription response that is not likely to be associated with any physiologically relevant functional adaptation. The sublethal concentrations did not generate a strong “functional” gene transcription signal, although a handful of genes could be linked to maintenance of homeostasis in spite of the disruption of Na^+^ import/export (swelling, osmotic stress). In particular, the differential downregulation of profilin associated with decreased sensitivity in PA21A092-treated cultures indicated a possible protective response that was absent in the KAE609 response (increased sensitivity).

Although many more altered metabolites were detected than gene transcripts, detailed interpretation of these changes are hampered by the interconnected and time-dependent nature of biochemical reactions and metabolites. A scattering of significant changes was noted among multiple metabolic pathways, and the largest and most consistent difference between PA21A092 and KAE609 treated cultures was potentially related to a differential response to Na^+^ homeostasis disruption. To study treatment-induced disruption of the fine-tuned metabolic network aspect of malaria parasite metabolism, a genome-scale metabolic network modelling technique integrating the time-dependent transcriptomic and metabolomic data was used. This provides a functional metabolic description that is easier to interpret as it takes into account the coupled physiological response via the inter-connected network of biochemical reactions.

The overall stage-dependent syntheses of macromolecules characterize the parasite progression through the IDC from rings to schizonts. The model-predicted rates of macromolecular synthesis show the greatest deviation from the parental Dd2 line in the PA21A092-treated cultures, in particular for lipid synthesis, whereas the KAE609-treated cultures show far less deviations. A detailed analysis of the predicted reaction fluxes identified several coupled metabolic reactions that were predicted to be active only in the less sensitive PA21A092-treated cultures. These reactions connect several of the individual metabolites found from the metabolomic data with an overall process that links Na^+^ homeostasis disruption to an increased production of lipids. The overproduction of lipids appears to be a metabolism by-product to maintain overall vitality. Interestingly, and in spite of applying the same drug pressure (~ 50% reduction of parasite replication) using KAE609, the same shift in metabolism was not observed. These results highlight subtle differences in parasite metabolism that drive the response to sublethal drug exposures.

## Supplementary Information

**Additional file 1:Figure S1.** Experiments determining sublethal doses of PA21A092 and KAE609.

**Additional file 2: Figure S2.** Effect of PA21A092 and KAE609 on daughter merozoites per schizont (mature parasite).

**Additional file 3: Figure S3.** Parasitemia in Dd2^A211V^ cultures at 400 nM PA21A092 and 30 pM KAE609.

**Additional file 4: Figure S4.*** De novo *myoinositol synthesis in *P. falciparum*.

**Additional file 5: Figure S5.** Expression of genes encoding proton pyrophosphatases in *P. falciparum*.

**Additional file 6: Table S1.** Raw metabolomic data obtained from parasite-infected red blood cell cultures and uninfected red blood cell cultures maintained in the presence of pure RPMI medium containing a sublethal dose of KAE609 (Sheet 1) and PA21A092 (Sheet 2).

**Additional file 7: Table S2.** Significantly altered genes in KAE609 (Sheet 1) and PA21A092 (Sheet 2) treated Dd2^A211V^ parasites, shown in Fig. 2C of the manuscript.

**Additional file 8: Table S3.** Metabolic reactions associated with differential metabolism of KAE609- and PA21A092-treated parasites (discussed with Fig. 5).

**Additional file 9: Table S4.** ToxProfiler results (Sheet 1) and expression of *Plasmodium* genes orthologus to ABCB1 gene (Sheet 2).

## Data Availability

All data generated or analysed during this study are included in this published article and its supplementary information files.
