# Supplementary material for: Metabolic responses in blood-stage malaria parasites associated with increased and decreased sensitivity to PfATP4 inhibitors
Source: Malar J. 2023 Feb 14;22:56. doi: 10.1186/s12936-023-04481-x (PMC9930341; doi:10.1186/s12936-023-04481-x)
Supplement: Supplementary file 1 — Additional file 1:Figure S1. Experiments determining sublethal doses of PA21A092 and KAE609. [file 12936_2023_4481_MOESM1_ESM.pptx]

## Slide 1
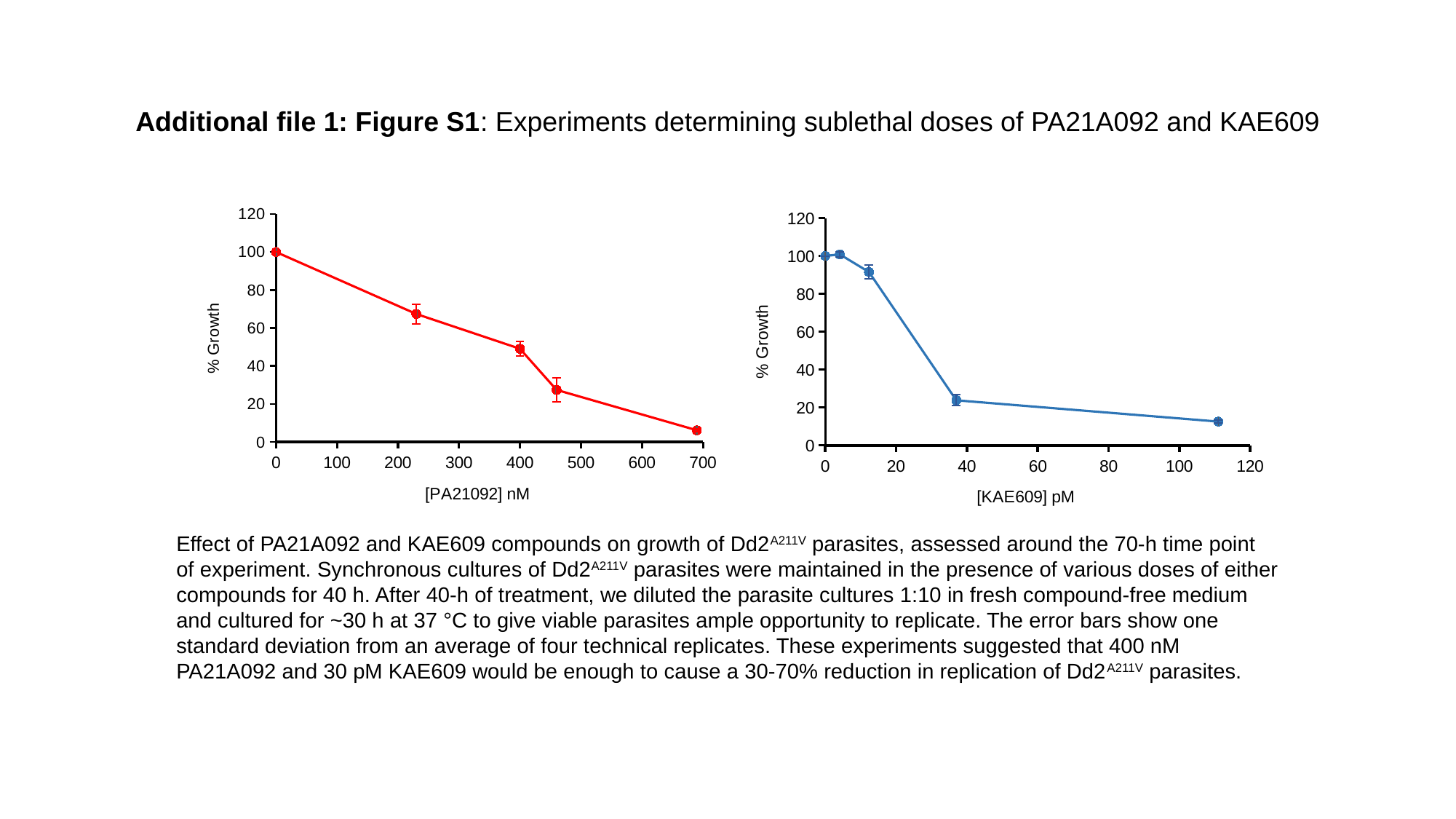

# Additional file 1: Figure S1: Experiments determining sublethal doses of PA21A092 and KAE609
### Chart
| Category | Average |
|---|---|
### Chart
| Category | Average |
|---|---|Effect of PA21A092 and KAE609 compounds on growth of Dd2A211V parasites, assessed around the 70-h time point of experiment. Synchronous cultures of Dd2A211V parasites were maintained in the presence of various doses of either compounds for 40 h. After 40-h of treatment, we diluted the parasite cultures 1:10 in fresh compound-free medium and cultured for ~30 h at 37 °C to give viable parasites ample opportunity to replicate. The error bars show one standard deviation from an average of four technical replicates. These experiments suggested that 400 nM PA21A092 and 30 pM KAE609 would be enough to cause a 30-70% reduction in replication of Dd2A211V parasites.
