# Supplementary material for: Metabolic responses in blood-stage malaria parasites associated with increased and decreased sensitivity to PfATP4 inhibitors
Source: Malar J. 2023 Feb 14;22:56. doi: 10.1186/s12936-023-04481-x (PMC9930341; doi:10.1186/s12936-023-04481-x)
Supplement: Supplementary file 2 — Additional file 2: Figure S2. Effect of PA21A092 and KAE609 on daughter merozoites per schizont (mature parasite). [file 12936_2023_4481_MOESM2_ESM.pptx]

## Slide 1
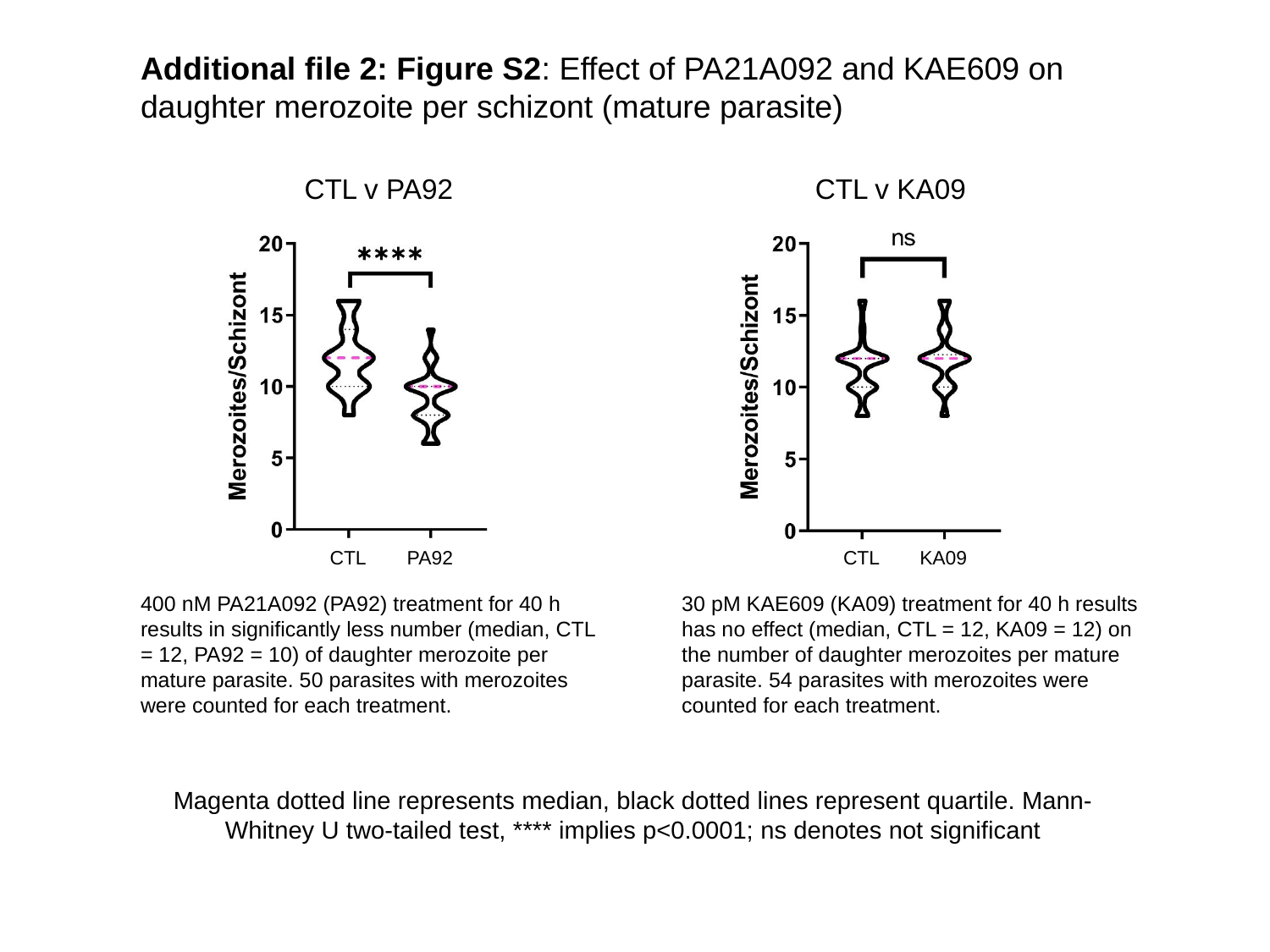

Additional file 2: Figure S2: Effect of PA21A092 and KAE609 on daughter merozoite per schizont (mature parasite)
CTL v PA92
CTL v KA09
CTL
PA92
CTL
KA09
400 nM PA21A092 (PA92) treatment for 40 h results in significantly less number (median, CTL = 12, PA92 = 10) of daughter merozoite per mature parasite. 50 parasites with merozoites were counted for each treatment.
30 pM KAE609 (KA09) treatment for 40 h results has no effect (median, CTL = 12, KA09 = 12) on the number of daughter merozoites per mature parasite. 54 parasites with merozoites were counted for each treatment.
Magenta dotted line represents median, black dotted lines represent quartile. Mann-Whitney U two-tailed test, **** implies p<0.0001; ns denotes not significant
