# Supplementary material for: Metabolic responses in blood-stage malaria parasites associated with increased and decreased sensitivity to PfATP4 inhibitors
Source: Malar J. 2023 Feb 14;22:56. doi: 10.1186/s12936-023-04481-x (PMC9930341; doi:10.1186/s12936-023-04481-x)
Supplement: Supplementary file 3 — Additional file 3: Figure S3. Parasitemia in Dd2A211V cultures at 400 nM PA21A092 and 30 pM KAE609. [file 12936_2023_4481_MOESM3_ESM.pptx]

## Slide 1
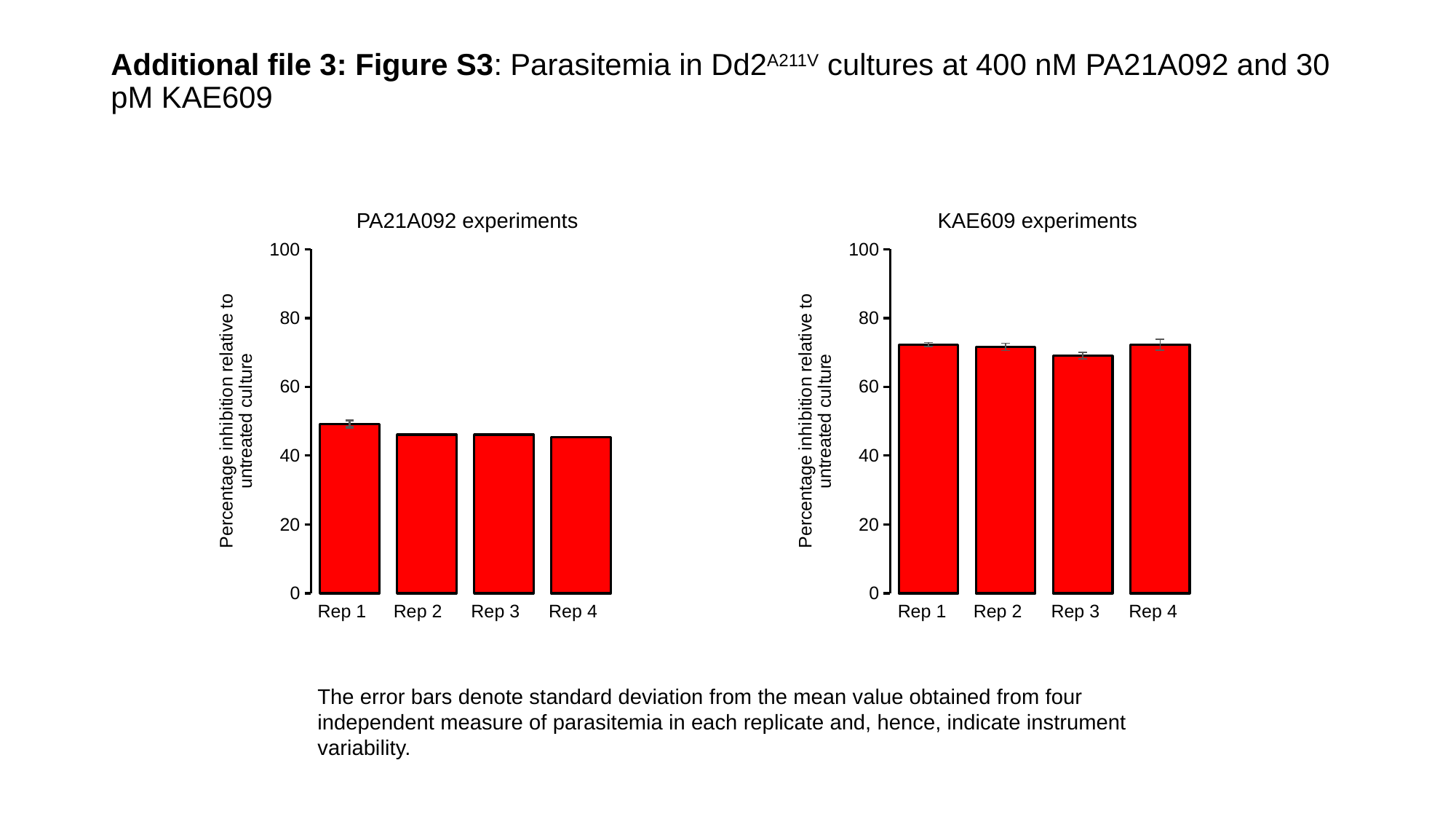

# Additional file 3: Figure S3: Parasitemia in Dd2A211V cultures at 400 nM PA21A092 and 30 pM KAE609
KAE609 experiments
PA21A092 experiments
### Chart
| Category | |
|---|---|
| Rep 1 | 49.239851828985955 |
| Rep 2 | 46.11436950146627 |
| Rep 3 | 46.114369501466285 |
| Rep 4 | 45.361938570767094 |
### Chart
| Category | |
|---|---|
| Flask 1 | 72.31727069561474 |
| Flask 2 | 71.64756156671265 |
| Flask 3 | 69.04935979514806 |
| Flask 4 | 72.30719399132687 |Rep 3
Rep 4
Rep 3
Rep 4
Rep 2
Rep 2
Rep 1
Rep 1
The error bars denote standard deviation from the mean value obtained from four independent measure of parasitemia in each replicate and, hence, indicate instrument variability.
