# Supplementary material for: Metabolic responses in blood-stage malaria parasites associated with increased and decreased sensitivity to PfATP4 inhibitors
Source: Malar J. 2023 Feb 14;22:56. doi: 10.1186/s12936-023-04481-x (PMC9930341; doi:10.1186/s12936-023-04481-x)
Supplement: Supplementary file 4 — Additional file 4: Figure S4. De novo myoinositol synthesis in P. falciparum. [file 12936_2023_4481_MOESM4_ESM.pptx]

## Slide 1
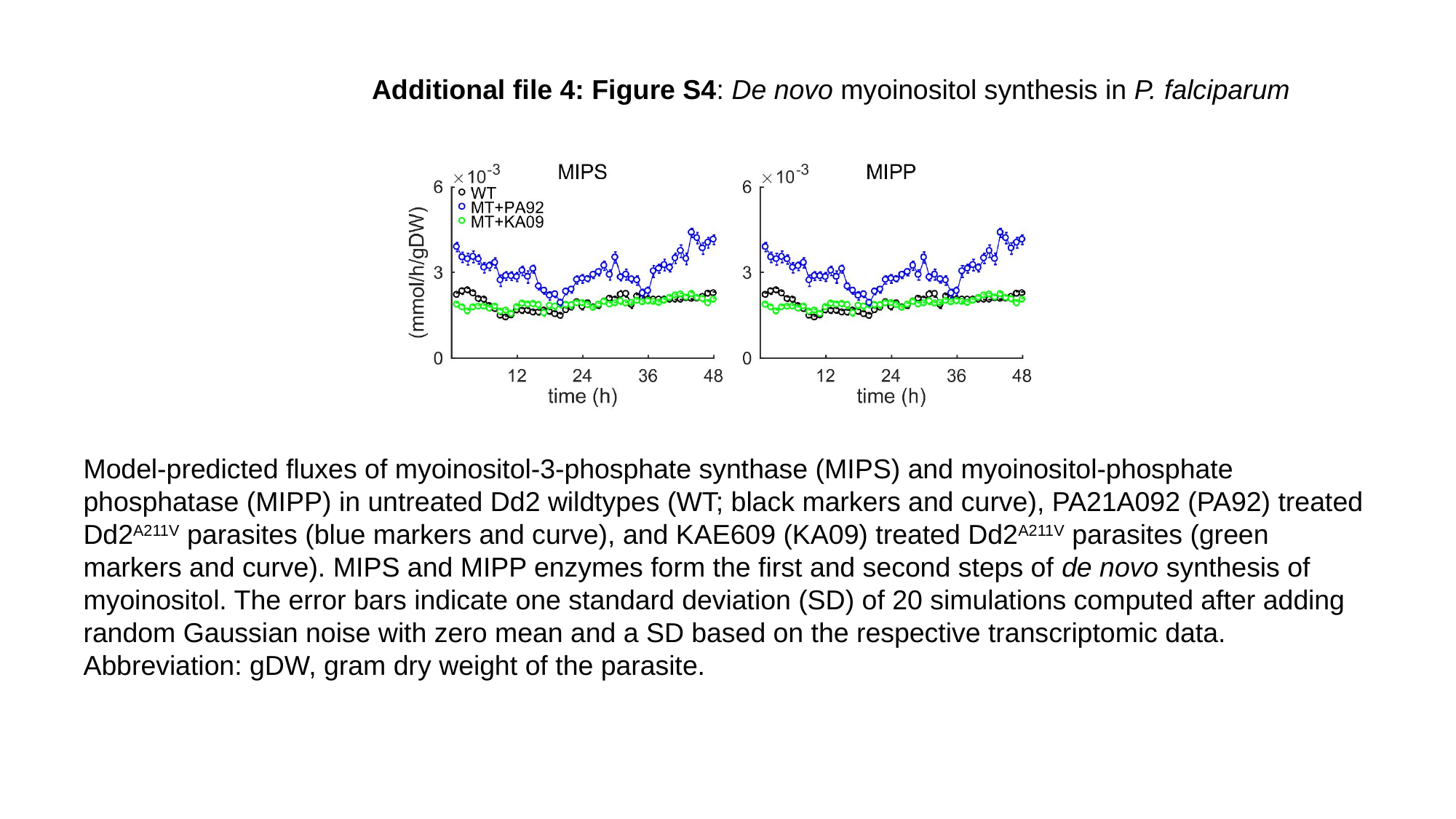

Additional file 4: Figure S4: De novo myoinositol synthesis in P. falciparum
Model-predicted fluxes of myoinositol-3-phosphate synthase (MIPS) and myoinositol-phosphate phosphatase (MIPP) in untreated Dd2 wildtypes (WT; black markers and curve), PA21A092 (PA92) treated Dd2A211V parasites (blue markers and curve), and KAE609 (KA09) treated Dd2A211V parasites (green markers and curve). MIPS and MIPP enzymes form the first and second steps of de novo synthesis of myoinositol. The error bars indicate one standard deviation (SD) of 20 simulations computed after adding random Gaussian noise with zero mean and a SD based on the respective transcriptomic data. Abbreviation: gDW, gram dry weight of the parasite.
