# Supplementary material for: Metabolic responses in blood-stage malaria parasites associated with increased and decreased sensitivity to PfATP4 inhibitors
Source: Malar J. 2023 Feb 14;22:56. doi: 10.1186/s12936-023-04481-x (PMC9930341; doi:10.1186/s12936-023-04481-x)
Supplement: Supplementary file 5 — Additional file 5: Figure S5. Expression of genes encoding proton pyrophosphatases in P. falciparum. [file 12936_2023_4481_MOESM5_ESM.pptx]

## Slide 1
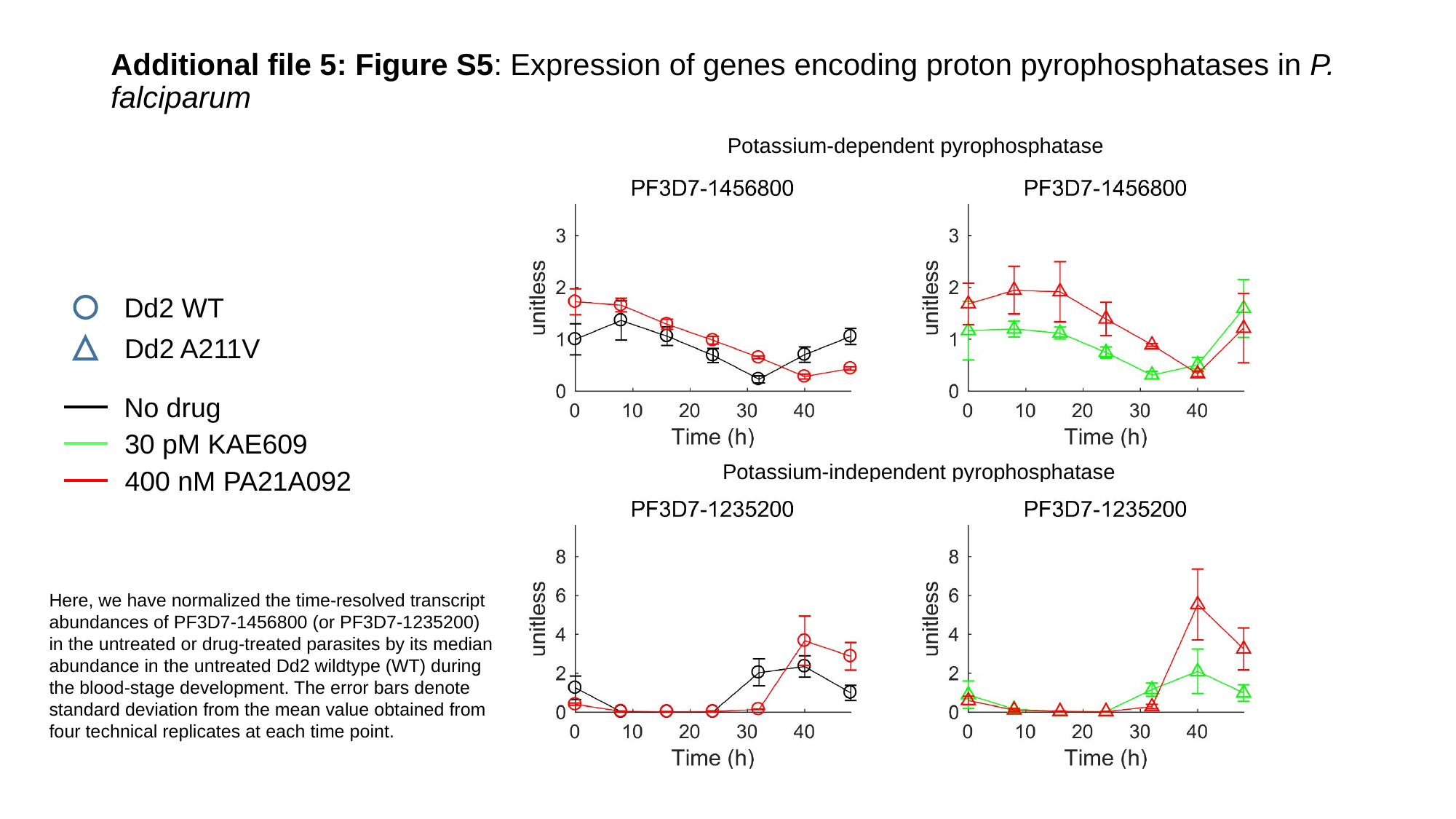

# Additional file 5: Figure S5: Expression of genes encoding proton pyrophosphatases in P. falciparum
Potassium-dependent pyrophosphatase
Dd2 WT
Dd2 A211V
No drug
30 pM KAE609
Potassium-independent pyrophosphatase
400 nM PA21A092
Here, we have normalized the time-resolved transcript abundances of PF3D7-1456800 (or PF3D7-1235200) in the untreated or drug-treated parasites by its median abundance in the untreated Dd2 wildtype (WT) during the blood-stage development. The error bars denote standard deviation from the mean value obtained from four technical replicates at each time point.
